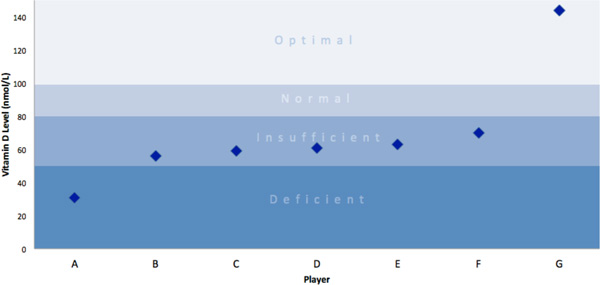# Observational case study - Vitamin 25(OH)D status of professional basketball players and its impact on athletic performance and recovery

**DOI:** 10.1186/1550-2783-12-S1-P55

**Published:** 2015-09-21

**Authors:** Marc Bubbs

**Affiliations:** 1 Sports Nutrition Lead, Canada Basketball, 1 Westside Drive, Suite 11, Etobicoke, ON, M9C 1B2, Canada

## Background

The rate of vitamin D insufficiency is estimated at greater than three-quarters of the general population and therefore it's likely many athletes fall into this same category. Vitamin D's role in calcium regulation and bone health is well documented, however new research highlights vitamin D's potential role in athletic performance and recovery via its potential impact on protein synthesis, muscle function, hormone synthesis, immune response, inflammation and regulation of lean muscle.

## Objective

To highlight the prevalence of insufficient serum vitamin 25(OH)D levels in professional basketball players training at high-intensity and its potential impact on performance and recovery.

## Methods

Serum vitamin 25(OH)D levels were collected at pre-training camp medicals for 7 of the 12 players on the Canadian Men's Olympic Basketball team. Collection was done via blood draw the day before training camp started (mid-July 2014), after the conclusion of the athlete's competitive season.

## Results

The serum vitamin 25(OH)D levels for the seven players measured in nmol/L at training were as follows; 31, 56, 59, 61, 63, 70, and 144 nmol/L. The mean serum vitamin 25(OH)D results for the seven players tested was 69 nmol/L, while the median score was 61 nmol/L.

## Discussion

The optimal serum level of vitamin 25(OH) D has not been established, however vitamin D deficiency is typically defined as < 50 nmol/L ( < 20 ng/mL), insufficiency defined as 50-80 nmol/L (20-32 ng/mL), and optimal levels 100 nmol/L (> 40 ng/mL). It has been noted that at levels < 40 ng/mL (100 nmol/L), the body relies on daily replenishment of vitamin 25(OH)D to meet its requirements and it's difficult to obtain this amount in the average diet.

The research available to support vitamin D's ability to increase performance is very limited, showing possible benefit in muscular strength, sprinting capacity, and VO_2 _max. Increased levels of inflammation from intense training (aerobic) have also been associated with low vitamin D levels. Vitamin D plays a key role in active muscle, as well as preventing stress fractures, supporting the notion that correcting vitamin D insufficiency may improve future performance. The direct cause of low or insufficient vitamin status in athletes training a high intensity is not clear and is most likely multi-factorial, due to inflammatory processes, muscular damage, increased protein synthesis requirements, increased immune activity, race, genetics or other unknown causes. Athletes competing in indoor sports may be at higher risk. (Note - player 'G' whose levels were 144 nmol/L was supplementing with vitamin D at the time of the assessment. He was the only player supplementing at the time of assessment).

## Conclusion

Athletes training at high-intensity seem more likely to have insufficient levels of vitamin 25(OH)D. The research in this area recommends athletes achieve > 40 ng/mL (100 nmol/L) to support overall health and athletic performance. These levels seem difficult to achieve without supplementation.

## Limitations & Future Considerations

In the future, obtaining values for all players on the roster and correcting for race, gender, disease, etc would help in further understanding the role of vitamin D in athletic performance and recovery.

**Figure 1:**